# A Behavioral-Genetic Perspective on Children of Alcoholics

**Published:** 1997

**Authors:** Matt McGue

**Affiliations:** Matt McGue, Ph.D., is a professor and associate chair in the Department of Psychology, University of Minnesota, Minneapolis, Minnesota

**Keywords:** children of alcoholics, hereditary vs. environmental factors, family environment, AOD use behavior, AODU (alcohol and other drug use) development, AODD (alcohol and other drug dependence), gender differences, risk factors, AOD dependence, adoption study, twin study, genetic markers, genotype, parent, behavioral and mental disorder, scientific model, literature review

## Abstract

Resemblance between parents and their children with respect to certain behaviors (e.g., alcohol use) may result from shared genes or from environmental influences that affect all members of a family similarly. Behavioral geneticists have used adoption, twin, and genetic marker studies to investigate the contributions of genetic as well as shared and nonshared environmental influences to the increased risk for alcoholism in children of alcoholics (COA’s). These analyses have found that in male COA’s, genetic makeup (i.e., genotype) plays an important role in the development of alcoholism; in female COA’s, however, the results were less consistent. Moreover, for both men and women, genetic factors alone cannot account for their risk of alcoholism. The behavioral-genetic concepts of genotype-environment interaction and genotype-environment correlation may provide useful models for the joint influences of genetic and environmental factors in the development of alcoholism.

Family studies have consistently demonstrated that many children of alcoholics (COA’s) exhibit a wide range of characteristics associated with their parents’ alcoholism.^1^ For example, compared with children of nonalcoholics (non-COA’s), COA’s are more likely to be alcoholic themselves (Cotton 1979) and to have other behavioral and psychiatric problems (West and Prinz 1987; Sher and Trull 1994). Moreover, COA’s frequently show more extreme manifestations than do non-COA’s of the temperament characteristics associated with an increased risk for alcoholism (e.g., behavioral disinhibition and negative emotionality) (Sher 1991). Although the prognostic significance of parental alcoholism on COA functioning is undisputed, researchers do not yet fully understand the causes and mechanisms underlying these parent-offspring correlations.

Any behavioral resemblance among members of an intact nuclear family (i.e., biological mother, biological father, and children) may result from shared genes or from the influences of their shared, or common, environment. Thus, the observation that COA’s exhibit elevated rates of behavioral disorders does not indicate whether this increased risk results from the genes that alcoholic parents transmit to their children, some deficiency in the rearing environment provided by alcoholic parents, or a combination of both of these influences. Unfortunately, when interpreting findings from studies on COA’s, researchers have not always taken into account this fundamental limitation of family study methods. Accordingly, biologically oriented researchers tend to interpret familial associations as reflecting biological mechanisms, whereas psychosocially oriented researchers generally consider familial associations the result of environmental mechanisms.

This article reviews findings of behavioral-genetic research on alcoholism, including the results of both adoption and twin studies. These analyses indicate that genetic factors play a major role in the relationship between parental alcoholism and COA functioning. The environmental influences resulting from being reared by an alcoholic parent, in contrast, do not appear to increase the risk of alcoholism in the children. This article also discusses two models—genotype-environment interaction and genotype-environment correlation—that may explain how a person’s genetic makeup (i.e., genotype) and environment jointly influence the person’s risk of alcoholism.

## Methods of Behavioral Genetics

**Figure 1:**
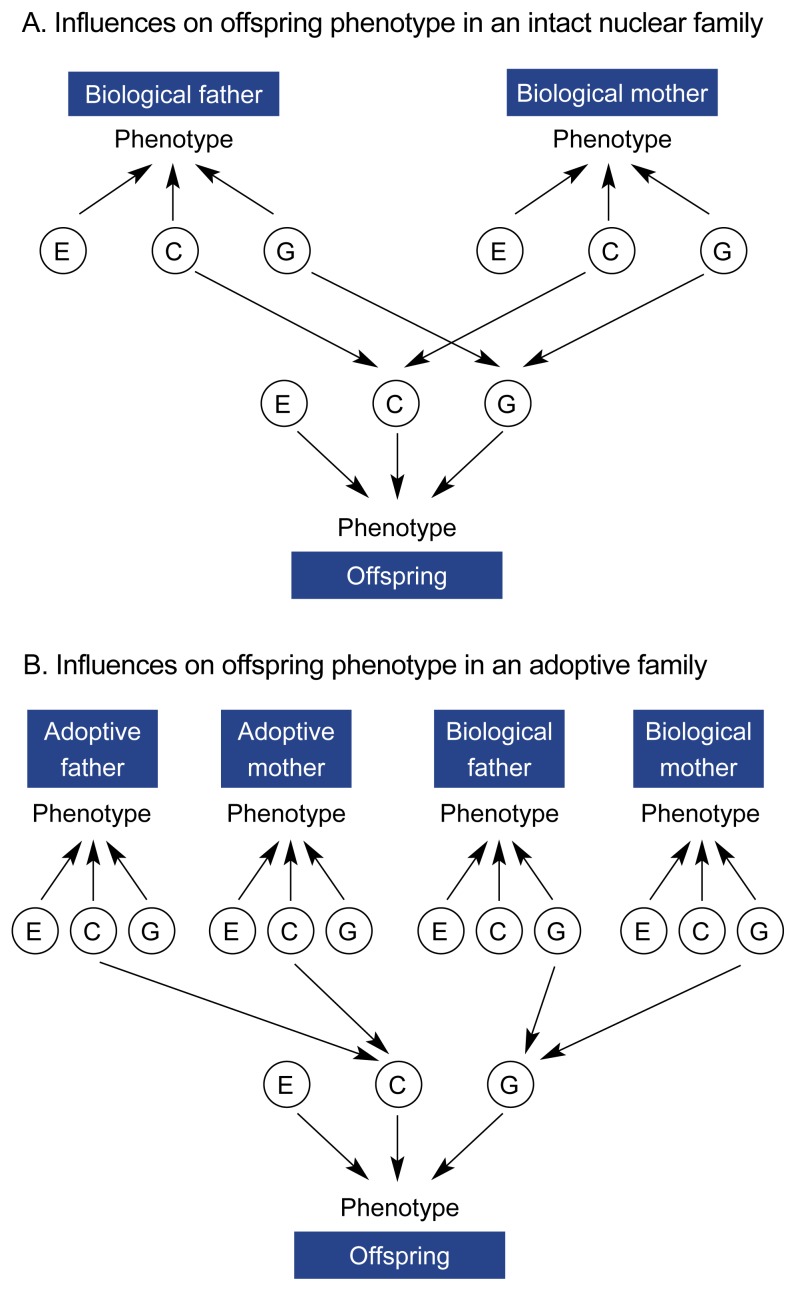
Contributions of genetic (G), shared—or common—environmental (C), and nonshared environmental (E) factors to the resemblance in phenotype (i.e., observable characteristics) between parents and offspring in intact nuclear and adoptive families. (A) In intact nuclear families, both genetic and shared environmental factors can contribute to parent-offspring resemblance with respect to a phenotype, such as alcoholism. (B) In adoptive families, genetic factors contribute to the resemblance between the offspring and the biological parents, whereas shared environmental factors contribute to the resemblance between the offspring and the adoptive parents.

Behavioral geneticists distinguish three major contributors to individual differences in people’s observable characteristics (i.e., phenotypes): genetic factors, shared environmental factors, and nonshared environmental factors. Shared, or common, environmental factors equally affect all members of a group of people living together and are therefore a potential source of behavioral similarities among these people. Examples of shared environmental factors include parental child-rearing strategies, the environmental consequences of parental psychopathology (e.g., alcoholism), and family income and social status. Nonshared environmental factors are not experienced by all members of a group living together and therefore may contribute to behavioral differences. For example, differential treatment by parents, different peer groups, and idiosyncratic traumatic events constitute nonshared environmental factors. In intact nuclear families, both shared environmental factors and genetic factors can contribute to parent-offspring resemblance (see figure 1). To assess the influences of all these factors on a certain behavior, behavioral geneticists use several research approaches, including adoption studies, twin studies, and genetic marker studies.

### Adoption Studies

The most direct approach for determining the respective contributions of genetic and shared environmental factors to parent-child resemblance in intact nuclear families is the analysis of adoptive families. A person who has been adopted as an infant by biologically unrelated parents will, in principle, share only genetic factors with her or his biological relatives but will share common environmental factors with her or his adoptive relatives (see figure 1). In practice, several factors can mitigate the clean separation of these two sources of familial resemblance. For example, the prenatal environment is affected by the biological parents (e.g., by prenatal alcohol exposure) and is thus a potential source of environmental resemblance between birth parents and their adopted-away children. These influences originate principally from the mother, although paternal contributions are also possible. In addition, in some cases, matching occurs on characteristics of the biological parents with characteristics of the adoptive parents (i.e., selective placement). This process could induce a correlation between genetic factors and the rearing environment if, for example, COA’s were preferentially placed with adoptive parents who have behavioral problems. Little evidence exists, however, for selective placement with respect to characteristics relevant to the development (i.e., etiology) of alcoholism.

### Twin Studies

Alternative approaches, such as twin studies, can overcome some of the limitations of adoption studies. Twin studies compare the similarities between identical (i.e., monozygotic [MZ]) twins who have been reared together with the similarities between fraternal (i.e., dizygotic [DZ]) twins who have been reared together. Whereas MZ twins share all their genes, DZ twins, like ordinary siblings, share on average only 50 percent of their genes. Because twin births occur relatively frequently,^2^ and because the Scandinavian countries and some U.S. States maintain population-based twin registries, twin studies have been an especially popular approach for investigating genetic and environmental influences on the etiology of alcoholism and other complex behavioral disorders.

For categorical phenotypes such as alcoholism, which occur only in a few distinct manifestations, twin similarity is usually assessed using the concordance statistic, which is the rate of alcoholism among co-twins of alcoholic twins. Because twins generally are assumed to share all environmental influences, findings of greater phenotypic similarities between MZ than between DZ twins imply the existence of significant genetic influences. Conversely, equal similarities between MZ and DZ twins suggest that the phenotype studied is primarily determined by environmental influences. Various mathematical models allow more specific estimates of the contributions of genetic, shared environmental, and nonshared environmental factors influencing the behavior studied (see box, p. 213).

**Calculating the Contributions of Genetic and Environmental Factors**

Scientists have developed several mathematical models to determine the contribution of genetic factors (i.e., heritability), shared environmental factors, and nonshared environmental factors to a given behavior based on the concordance of this behavior in identical (i.e., monozygotic [MZ]) and fraternal (i.e., dizygotic [DZ]) twins. These formulas rely on previously determined correlation coefficients, which are numerical ways of describing the relation of the behavior under study to the environmental or genetic factors hypothesized to underlie that behavior. A correlation coefficient of 1 describes a perfect positive correlation (e.g., the presence of a particular gene means that a particular behavior, such as problem drinking, will almost certainly be seen in the subject). A correlation of 0 means that no relationship exists between the hypothesized causal factor and the behavior, and a correlation of −1 means that the presence of the environmental or genetic factor almost certainly indicates the absence of the behavior (i.e., a perfect negative correlation).

In the simplest model, heritability is estimated as twice the difference between the correlation coefficient for the relation of genetic factors to alcoholism in MZ twins and the correlation in DZ twins (i.e., 2[MZ–DZ]). Thus, if the correlation for alcoholism risk is 0.7 for MZ twins and 0.4 for DZ twins, the heritability would be 0.6 (i.e., 2[0.7–0.4]).

The contribution of shared environmental factors is defined as the difference between the correlation in MZ twins and the heritability estimate calculated above. Using the same MZ and DZ correlation coefficients, the role of shared environmental factors in alcoholism can be described as 0.7–0.6, or 0.1.

Finally, the contribution of nonshared environmental factors is calculated as the difference between 1.0 and the correlation in MZ twins. With the correlation coefficients given in the previous examples, the role of nonshared environmental factors is 1–0.7, or 0.3.

For many studies (e.g., if the numbers of MZ and DZ twin pairs in the study differ greatly), however, these simple calculations are inadequate, and more complex models must be used to estimate the contributions of all three types of factors to the behavior studied. The results of such analyses have consistently found that the heritability of the risk for alcoholism lies between 0.5 and 0.6 for men. The findings for women have been much less consistent.

The assumption that MZ and DZ twins share environmental influences to the same extent (i.e., the equal-environmental similarity assumption), however, may not always be accurate. For example, the two members of a twin pair can have different groups of friends or be treated differently by their parents. If these differences are more common among DZ than among MZ twins, a twin study will interpret this to be a genetic rather than an environmental effect.

### Genetic Marker Studies

As discussed in the following sections, both adoption and twin studies have indicated that one or more of the approximately 50,000 to 100,000 genes comprising the human genetic material (i.e., the human genome) influence a person’s likelihood of developing alcoholism. Increasingly, alcohol researchers seek to confirm the existence of these genetic influences by identifying the specific genes involved using molecular genetic methods. This effort has been greatly facilitated by the Human Genome Project, an international research effort to decipher the complete human genome (Collins and Fink 1995). This project already has produced a so-called linkage map of the genome, which consists of thousands of well-characterized landmark genetic sequences (i.e., markers) that are dispersed throughout the genome and which can be used to determine the locations of disease-susceptibility genes.

Because of the complexity of behavioral phenotypes, such as alcoholism, however, progress in identifying relevant genes has been slow. Apart from the well-known gene products involved in alcohol metabolism, no gene products have been associated unequivocally with the risk for alcoholism. If findings from adoption and twin studies of alcoholism are valid, however, replicable associations of alcoholism with specific genetic markers should emerge over the next 5 to 10 years.

## Genetic Contributions to Parent-Offspring Resemblance for Alcoholism

Several adoption and twin studies have analyzed the contribution of genetic factors to a person’s risk for alcoholism. For example, five adoption studies have investigated the relationship between alcoholism or problem drinking in biological parents and alcoholism in their adopted-away sons. With the exception of a small early study, all of these studies found a significant correlation between alcoholism in male adoptees and their biological parents. Thus, the rate of alcoholism was two to three times higher in adopted-away sons of alcoholics than in adopted-away sons of nonalcoholics (McGue 1995).

The largest published adoption study of alcoholism, the Stockholm Adoption Study (Sigvardsson et al. 1996), illustrates these findings. The study included 862 men born in Stockholm, Sweden, who had been adopted in infancy and both their biological and adoptive parents. Alcohol abuse among the study participants was assessed by the number of registrations with local temperance boards—agencies in each Swedish community that document how often a person has been cited or treated for alcohol abuse. In this study, the rate of alcohol abuse among the adoptees was 14.7 percent if neither biological parent abused alcohol, 22.4 percent if only the biological father abused alcohol, 26.0 percent if only the biological mother abused alcohol, and 33.3 percent if both biological parents abused alcohol. Thus, adoptees with one or two alcohol-abusing parents had a significantly greater risk of alcohol abuse than did adoptees with no alcohol-abusing parents.

Analyses of reared-together male twins have supported the findings from the adoption studies. Again, with the exception of one small study, the six published studies of alcoholism in male twins found 50- to 200-percent greater concordance rates for alcoholism among MZ than among DZ twins (McGue 1995). For example, in one study of 86 pairs of male twins, the concordance rate for alcohol dependence^3^ was 59 percent among MZ twins but only 36 percent among DZ twins (Pickens et al. 1991).

Adoption and twin studies of alcoholism in men have consistently implicated genetic factors in the development of the disorder, whereas behavioral-genetic research on alcoholism in women has produced seemingly inconsistent findings. Only two of four adoption studies have reported a significant correlation between alcoholism in female adoptees and their biological parents, and only two of five twin studies found significantly greater concordance among female MZ twins than among female DZ twins (McGue 1995). These results have led several researchers to hypothesize that genetic factors are less important to the etiology of alcoholism in women than in men (e.g., McGue and Slutske 1996).

The most recent and best designed adoption and twin studies that included women, however, observed significant genetic effects on the alcoholism risk in their subjects. For example, in the Stockholm Adoption Study, which also is the largest adoption study for women, the rate of alcohol abuse was significantly greater among the adopted-away daughters of alcohol-abusing mothers (i.e., 9.8 percent) than among the adopted-away daughters of non-alcohol-abusing mothers (i.e., 2.8 percent) (Bohman et al. 1981). Conversely, paternal alcohol abuse did not affect the adopted-away daughters’ risk of alcohol abuse, suggesting that genetic effects might be gender-specific. Finally, the largest twin study of women found that concordance for alcoholism was significantly greater among MZ than among DZ twins (i.e., 32 and 24 percent, respectively) (Kendler et al. 1994).

The apparently inconsistent pattern of results in adoption and twin studies of alcoholism in women may be attributed to the relatively small sample sizes that characterize research in this area and allow only limited statistical analyses. Despite great variations between studies in the estimates of the contribution of genetic factors to alcoholism in women, the confidence intervals associated with these estimates are wide and overlapping^4^ (Heath et al. 1997), suggesting that genetic factors exert some influence on women’s risk of alcoholism. Nevertheless, the existing research does not allow one either to determine precisely the magnitude of that influence or to calculate whether it is comparable to or weaker than the influence of genetic factors on men’s risk of alcoholism.

Inconsistencies with respect to the contribution of genetic factors among various studies in men and women also may arise from the existence of different types of alcoholism that may be differentially heritable. For example, Cloninger (1987) has hypothesized that two types of alcoholism exist: type I and type II. Type I alcoholism affects both men and women, is characterized by relatively late onset, and is rarely associated with antisocial behavior. Conversely, type II alcoholism primarily affects men, is characterized by relatively early onset, and is frequently associated with antisocial behavior.

The results of both adoption and twin studies have suggested that genetic influences affect type II alcoholism more strongly than type I alcoholism. For example, in the Stockholm Adoption Study, genetic factors were estimated to account for 90 percent of the risk for type II alcoholism, but for less than 40 percent of the risk for type I alcoholism (Cloninger et al. 1981). Similarly, in one twin study, the difference in concordance between MZ and DZ twins was greater for twin pairs with early onset (type II) alcoholism than for those with late onset (type I) alcoholism (McGue 1995).

## Environmental Contributions to Parent-Offspring Resemblance for Alcoholism

Although both adoption and twin studies consistently have indicated that, at least in men, genetic factors contribute to parent-offspring resemblance for alcoholism, these factors may not account for the entire increase in the risk for alcoholism among COA’s. Conceivably, environmental liabilities associated with being reared by an alcoholic parent also can increase a person’s risk for alcoholism. To address this issue, researchers have investigated the rate of alcoholism among adoptees with nonalcoholic biological parents who have been reared by alcoholic adoptive parents. In these cases, no genetic contributions exist to the resemblance between adoptive parent and adoptee. Alcoholism is relatively rare among adoptive parents, who usually must undergo some type of informal mental health screening before being accepted for placement. Enough cases of alcoholic adoptive parents occur, however, to allow researchers to determine whether being reared by a nonbiological alcoholic parent increases a person’s risk for alcoholism.

Both Danish and Swedish adoption studies found that alcoholism rates were not significantly higher among adoptees reared in families with an alcoholic adoptive parent compared with adoptees reared by nonalcoholic adoptive parents. For example, in the Stockholm Adoption Study, the rates of alcohol abuse were 13 percent among male adoptees reared in a home with an alcohol-abusing parent and 18 percent among male adoptees reared in a home with no alcohol-abusing parent (Cloninger et al. 1981). The corresponding rates for female adoptees were 3.7 and 3.4 percent, respectively (Bohman et al. 1981).

In contrast, two studies conducted in the United States found that a history of alcoholism in an adoptive family was associated with adoptee alcoholism (Cadoret et al. 1985, 1987). Unlike the Scandinavian studies, which focused specifically on alcoholism among adoptive parents, the U.S. studies included a history of alcoholism among both adoptive parents and adoptive siblings. Consequently, the discrepancy between the findings of the Scandinavian and U.S. studies may reflect the differential impact of siblings and parents on a person’s risk for alcoholism. The findings suggest that siblings, rather than parents, may be the primary source of family environmental influences on alcoholism risk.

This hypothesis also is supported by the findings of a recent large adoption study of adolescent alcohol use (McGue et al. 1996). In this study, parental drinking was significantly related to adolescent drinking among biological but not adoptive children, again indicating that the parent-child resemblance for alcohol-related phenotypes is primarily mediated by genetic factors. Moreover, a significantly greater correlation existed between parental ratings of family climate (e.g., the cohesiveness of the family) and adolescent alcohol use in biological families compared with adoptive families, suggesting that genetic factors may also be a major contributor to this correlation. In sharp contrast to the lack of similarity in drinking behavior between adolescents and their adoptive parents was the statistically significant similarity between the adolescents and their adoptive siblings. The correlation among adoptive siblings for the amounts of alcohol consumed was 0.24 when all siblings were considered. Moreover, the correlation was higher (i.e., 0.45) among sibling pairs of the same sex and/or close in age, but lower (i.e., 0.05) among sibling pairs of opposite sex and/or distant in age.

Because adoptive sibling pairs are not biologically related, correlations between their behaviors must reflect environmental influences. Furthermore, the adoptive parents did not appear to be a source of these influences, because neither parental alcohol use nor parental ratings of the family climate were related to the adoptees’ alcohol use. Finally, the finding that alcohol use similarities among siblings were strongly influenced by the degree of demographic similarities among the siblings strongly suggests that siblings can be the primary source of familial environmental influence for alcohol-related phenotypes. Likewise, parents are not always the main source of the environmental factors that affect the development of alcoholism and alcohol-related phenotypes.

This conclusion may surprise many alcohol researchers, who, in focusing on parental environmental effects, may have neglected more potent sources of environmental influences. The findings are consistent, however, with a large body of behavioral-genetic research suggesting that the importance of parental environmental influences may have been overestimated in earlier research that failed to control for genetic contributions to parent-child resemblance (Plomin and Daniels 1987).

## Models for the Relative Contributions of Genetic and Environmental Factors

Behavioral-genetic research questions the nature, not the existence, of environmental influences on alcoholism risk. Both the finding that genetically identical MZ twins do not show 100-percent concordance in their drinking behavior and the inability to predict a person’s risk for alcoholism status based on the biological parents’ alcoholism status support the existence of substantial environmental influences on the etiology of alcoholism. Two behavioral-genetic concepts—genotype-environment interaction and genotype-environment correlation—are useful in modeling the joint influence of environmental and genetic factors on complex behavioral phenotypes such as alcoholism.

### Genotype-Environment Interactions

Not every person (i.e., not every genotype) is equally sensitive to environmental influences that affect a certain behavior. This differential sensitivity reflects genotype-environment interactions. One example of such interactions is the diathesis-stress model (see Windle, pp. 185–191), which is one of the most important concepts for explaining the development of any psychopathology. This model states that a person develops a given behavioral disorder only if he or she both inherits a vulnerability for the disorder (i.e., the diathesis) and is exposed to a provocative environment (i.e., the stress).

Behavioral-genetic researchers have attempted to identify genotype-environment interactions for alcoholism by using adoption studies. If the diathesis-stress model is a reasonable concept for explaining the development of alcoholism, then the risk of alcoholism among adopted-away COA’s (i.e., a group with a high genetic risk) should depend on their rearing environment. Conversely, the risk of alcoholism among adopted-away children of non-alcoholics (i.e., a group with a low genetic risk) should be largely independent of the rearing environment.

**Figure 2:**
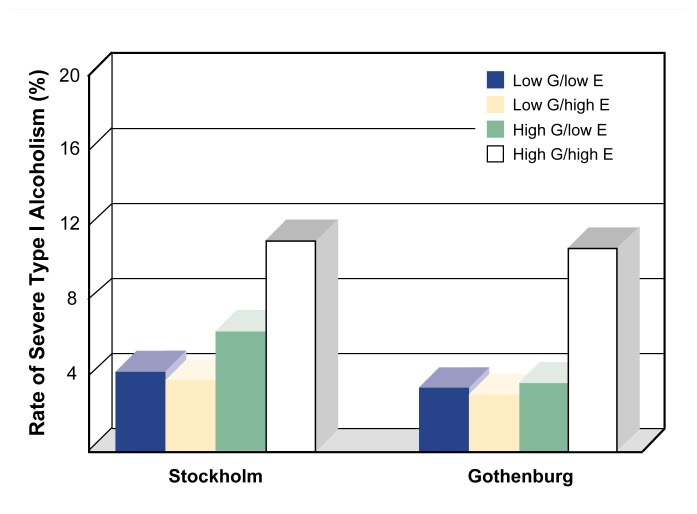
Rates of severe type I (i.e., late-onset) alcoholism among male participants in two adoption studies performed in Stockholm and Gothenburg, Sweden. Both the genetic (G) and the environmental (E) backgrounds of the adoptees were classified as either high or low risk based on multiple biological and environmental factors (e.g., the presence of type I alcoholism in the biological parents or the socioeconomic status of the adoptive family). Both studies found significantly elevated rates of severe type I alcoholism only among male adoptees who both had a high genetic risk and were reared in a high-risk environment. SOURCE: Adapted from Sigvardsson et al. 1996.

The strongest support for the diathesis-stress model for alcoholism comes from the Stockholm Adoption Study and a replication of that study performed in Gothenburg, Sweden (Sigvardsson et al. 1996). In both studies, the researchers classified male adoptees’ genetic and environmental backgrounds as either high or low risk for the less strongly heritable type I alcoholism. For example, adoptees with a biological parent with late onset alcoholism were classified as having a relatively high genetic risk for type I alcoholism. Similarly, adoptees who were reared in adoptive homes with low socioeconomic status were classified as having a relatively high environmental risk for type I alcoholism. Both studies found that among adoptees with low genetic risk, the rearing environment did not affect the rates of type I alcoholism (see figure 2). Among adoptees with a high genetic risk, however, the type of rearing environment was associated with the rate of alcoholism. Thus, type I alcoholism was significantly more common among men reared in a provocative, high-risk environment than in a low-risk environment.

The existence of genotype-environment interactions suggests that the effect of environmental influences on alcoholism depends on a person’s genotype. The effects of the rearing environment may be negligible for the vast majority of people who inherit none or few of the relevant genetic risk factors. In contrast, the alcoholism risk for people who inherit some genetic risk factors may be affected substantially by the rearing environment. Alcohol researchers now hope that the findings from the Human Genome Project will aid them in precisely determining a person’s genetic risk for alcoholism. This knowledge will then help scientists in the focused investigation of the role of environmental influences in the etiology of alcoholism.

### Genotype-Environment Correlations

The concept of genotype-environment correlation refers to the processes through which genetic and environmental effects on a behavioral phenotype, such as alcoholism, become correlated. Two mechanisms of genotype-environment correlations are especially relevant in alcohol research: evocative genotype-environment correlations and active genotype-environment correlations. The concept of evocative genotype-environment interactions is based on the assumption that a person’s social environment is, in part, determined by the reactions that her or his behavior evokes from others. For example, teachers and parents react much differently to an overly active, defiant child than to a passive and compliant one. Thus, a child’s genetically influenced activity level or degree of defiance consistently evokes specific reactions from others, thereby causing the development of an evocative genotype-environment correlation. Active genotype-environment correlations, in contrast, are based on the premise that in a permissive society, people have a certain degree of freedom in constructing their own experiences. They choose their own friends, decide how hard they will work in school, and select their own leisure pursuits from a cornucopia of possibilities. To the extent that these experiential choices are guided by genetically influenced traits and abilities, an active genotype-environment correlation will ensue.

Until recently, evocative and active genotype-environment correlations were more a matter of speculation than investigation. Although researchers considered genotype-environment correlations to be fundamentally important in the etiology of various behavioral phenotypes, few empirical tests of these correlations existed. Ge and colleagues (1996), however, recently demonstrated how genotype-environment correlations could be investigated using an adoption study approach. These researchers investigated the factors contributing to the etiology of antisocial behavior among children.

The study found that adoptee antisocial behavior was significantly related to the biological parents’ history of psychiatric disorders (i.e., alcohol and other drug abuse or dependence or antisocial personality disorder), confirming that a genetic influence on adolescent antisocial behavior exists. Surprisingly, however, a correlation also existed between the adoptees’ genetic background and certain aspects of the rearing environment (e.g., disciplinary practices). Thus, adoptees whose biological parents had a history of psychiatric disorders were more likely to have adoptive parents who were harsh and less nurturing than were adoptees whose biological parents had no such history. This correlation between genetic background and rearing environment was mediated by the adoptees’ antisocial behavior and hostility: Adoptees whose biological parents had a history of psychiatric disorder were more likely than control adoptees to be hostile and antisocial. In turn, hostile and antisocial adoptees were more likely than nonantisocial and nonhostile adoptees to have adoptive parents who engaged in harsh disciplinary practices. This relationship between adoptee hostility and parental harsh discipline was reciprocal, meaning that hostility evoked harsh discipline, which, in turn, evoked more hostility.

Although the phenotype analyzed by Ge and colleagues (1996) may not be of direct interest to alcohol researchers, the methods and findings from this study clearly illustrate how genotype-environment correlations might affect complex behavioral phenotypes. It remains to be determined whether similar genotype-environment correlational processes exist in the development of alcoholism. Moreover, the study illustrates the concept—now accepted by most behavioral geneticists—that twin and adoption studies establish the heritability, but not the genetic determination, of behavior. This means that although a person’s genotype may predispose the person to a certain disorder, it does not inevitably lead to the development of that disorder. Consequently, it would be wrong to conclude that the existence of a genetic influence on alcoholism implies that this disorder can be approached only as a biological phenomenon. The primary function of genes is far removed from the behavior they influence, and the gene-to-behavior pathway can be mediated by intervening physiological, psychological, and social processes. The challenge for alcohol researchers now is to integrate the knowledge about the social and cultural influences on drinking and alcoholism with the emerging knowledge about the genetic influences on these behaviors.

## Footnotes

1 The terms “alcoholism,” “alcohol dependence,” “alcohol abuse,” and “problem drinking” frequently overlap in the alcoholism literature; therefore, the terms are used in this article as they were used in the original studies cited.

2 The frequency of twin births is approximately 1 in 50 among blacks, 1 in 80 among whites, and 1 in 125 among Asians.

3 In this study, the diagnosis of alcohol dependence was established after interviews were conducted with the participants. The diagnosis was based on the criteria of the American Psychiatric Association’s *Diagnostic and Statistical Manual of Mental Disorders*, *Third Edition* (DSM–III).

4 Statistical analyses frequently cannot calculate the exact value of the variable being studied. Instead, they provide a range of values —the confidence interval—that contains the actual value of the variable with a certain likelihood (e.g., 95 percent). The less reliable a statistical analysis is (e.g., because only a few subjects are available), the greater the confidence interval generally is. For example, one analysis might find a heritability factor for alcoholism of 0.6, with a 95-percent confidence interval ranging from 0.4 to 0.8. A second analysis might determine a heritability factor of 0.4, with a 95-percent confidence interval of 0.3 to 0.5. Thus, for the two studies, the estimates of heritability differ, but the confidence intervals overlap.

## References

[b1-arhw-21-3-210] Bohman M, Sigvardsson S, Cloninger CR (1981). Maternal inheritance of alcohol abuse: Cross-fostering analysis of adopted women. Archives of General Psychiatry.

[b2-arhw-21-3-210] Cadoret RJ, O’Gorman T, Troughton E, Heywood E (1985). Alcoholism and antisocial personality: Interrelationships, genetic and environmental factors. Archives of General Psychiatry.

[b3-arhw-21-3-210] Cadoret RJ, Troughton E, O’Gorman TW (1987). Genetic and environmental factors in alcohol abuse and antisocial personality. Journal of Studies on Alcohol.

[b4-arhw-21-3-210] Cloninger CR (1987). Neurogenetic adaptive mechanisms in alcoholism. Science.

[b5-arhw-21-3-210] Cloninger CR, Bohman M, Sigvardsson S (1981). Inheritance of alcohol abuse: Cross-fostering analysis of adopted men. Archives of General Psychiatry.

[b6-arhw-21-3-210] Collins FS, Fink L (1995). The human genome project. Alcohol Health & Research World.

[b7-arhw-21-3-210] Cotton NS (1979). The familial incidence of alcoholism: A review. Journal of Studies on Alcohol.

[b8-arhw-21-3-210] Ge X, Conger RD, Cadoret RJ, Neiderhiser JM, Yates W, Troughton E, Stewart MA (1996). The developmental interface between nature and nurture: A mutual influence model of child antisocial behavior and parent behaviors. Developmental Psychology.

[b9-arhw-21-3-210] Heath AC, Slutske WS, Madden PAF, Wilsnack RW, Wilsnack SC (1997). Gender differences in the genetic contribution to alcoholism risk and to alcohol consumption patterns. Gender and Alcohol: Individual and Social Perspectives.

[b10-arhw-21-3-210] Kendler KS, Neale M, Heath AC, Kessler RC, Eaves LJ (1994). A twin-family study of alcoholism in women. Archives of General Psychiatry.

[b11-arhw-21-3-210] McGue M, Turner JR, Cardon LR, Hewitt JK (1995). Mediators and moderators of alcoholism inheritance. Behavioral Genetic Approaches in Behavioral Medicine.

[b12-arhw-21-3-210] McGue M, Slutske W, Howard JM, Martin SE, Mail PD, Hilton ME, Taylor ED (1996). The inheritance of alcoholism in women. Women and Alcohol: Issues for Prevention Research.

[b13-arhw-21-3-210] McGue M, Sharma A, Benson P (1996). Parent and sibling influences on adolescent alcohol use and misuse: Evidence from a U.S. adoption cohort. Journal of Studies on Alcohol.

[b14-arhw-21-3-210] Pickens RW, Svikis DS, McGue M, Lykken DT, Heston LL, Clayton PJ (1991). Heterogeneity in the inheritance of alcoholism: A study of male and female twins. Archives of General Psychiatry.

[b15-arhw-21-3-210] Plomin R, Daniels D (1987). Why are children in the same family so different from one another?. Behavior and Brain Sciences.

[b16-arhw-21-3-210] Sher K (1991). Children of Alcoholics: A Critical Appraisal of Theory and Research.

[b17-arhw-21-3-210] Sher KJ, Trull TJ (1994). Personality and disinhibitory psychopathology: Alcoholism and antisocial personality disorder. Journal of Abnormal Psychology.

[b18-arhw-21-3-210] Sigvardsson S, Bohman M, Cloninger CR (1996). Replication of the Stockholm Adoption Study of alcoholism: Confirmatory cross-fostering analysis. Archives of General Psychiatry.

[b19-arhw-21-3-210] West MO, Prinz RJ (1987). Parental alcoholism and childhood psychopathology. Psychological Bulletin.

